# Nickel ENMs Activate HIF-1α

**DOI:** 10.1289/ehp.119-a512a

**Published:** 2011-12-01

**Authors:** Rebecca Kessler

**Affiliations:** Rebecca Kessler is a science and environmental journalist based in Providence, RI.

The health record of nickel, a known carcinogen, is far from shiny. Now the metal’s reputation has been further tarnished with the discovery that nano-scale nickel triggers a cellular pathway linked to cancer much more effectively than larger particles do.^1^

Engineered nanomaterials (ENMs) typically measure less than 100 nm in at least one dimension. Their minuscule size gives them novel physical and biological properties, and, being manufactured in a wide range of chemical compounds and shapes, they have applications in numerous fields.^2^ Nickel nanoparticles are used in catalysts, sensors, energy storage devices, and other products, although they are not yet made in great quantity. “It’s essential to study the toxicological properties so we understand them before they become widely used,” says Jodie Pietruska, a postdoctoral researcher at Brown University in Providence, Rhode Island, who led the new study.

Nickel is a well-known occupational hazard.^3^ In rodent studies, nickel nanoparticles instilled in the trachea and lung caused greater toxicity and inflammation than larger particles,^4^^,^^5^^,^^6^ and inhaled nickel nanoparticles caused signs of vascular disease.^7^^,^^8^ Brown University pathologist Agnes Kane coauthored a 2007 paper showing that, compared with larger micron-scale nickel, nickel nanoparticles release nickel ions more rapidly, a mechanism characteristic of carcinogenic nickel compounds.^9^

To further connect the dots, Kane, Pietruska, and colleagues conducted a series of experiments comparing the behavior in human lung epithelial cells of nickel oxide and pure metallic-nickel nanoparticles with larger metallic-nickel microparticles.^1^ They detected nickel ions inside lung epithelial cells exposed to nickel nanoparticles but not to nickel microparticles. And they showed that exposure to the nanoparticles, but not the microparticles, activated the HIF-1α cellular pathway, which is thought to be involved in carcinogenesis and tumor progression.

The researchers also found differences in overt toxicity of the various forms of nickel, with the nanoparticles killing the lung epithelial cells quickly and the microparticles having little effect. Intriguingly, in toxicity as well as in ion release and activation of the HIF-1α〈 pathway, nickel oxide was much more active than metallic nickel. Pietruska and Kane speculate that metallic nickel may be the more carcinogenic nanoparticle, because it seems more likely to let cells survive long enough to develop cancer. More research is needed, ultimately in live animals, before nickel nanoparticles can definitively be said to cause cancer.

Vincent Castranova, coordinator of nanotoxicology research at the National Institute for Occupational Safety and Health, says the finding has implications for agents besides nickel and reinforces the prediction that many ENMs will have greater biological effects than their larger-form counterparts. “It’s an important finding, but it’s not a surprising finding,” he says. “It confirms what would have been our suspicions.”
